# Supplementary material for: Intelligent perceptual textiles based on ionic-conductive and strong silk fibers
Source: Nat Commun. 2024 Apr 17;15:3289. doi: 10.1038/s41467-024-47665-y (PMC11024123; doi:10.1038/s41467-024-47665-y)
Supplement: Supplementary file 1 — Supplementary Information [file 41467_2024_47665_MOESM1_ESM.pdf]

Supplementary Information

Intelligent perceptual textiles based on ionic-conductive and strong silk fibers

Haojie Lu[1], Yong Zhang[1], Mengjia Zhu[1], Shuo Li[1], Huarun Liang[1], Peng Bi[1], Shuai Wang[1], Haomin Wang[1], Linli Gan[1], Xun-En Wu[1], Yingying Zhang[1]*

[1] Key Laboratory of Organic Optoelectronics and Molecular Engineering of the Ministry of Education, Department of Chemistry, Tsinghua University, Beijing 100084, P. R. China.

* Corresponding author. E-mail: yingyingzhang@tsinghua.edu.cn

Table of Contents

Supplementary Figures 1 to 21

Supplementary Tables 1 to 3

Supplementary references

Supplementary Figures 1 to 21

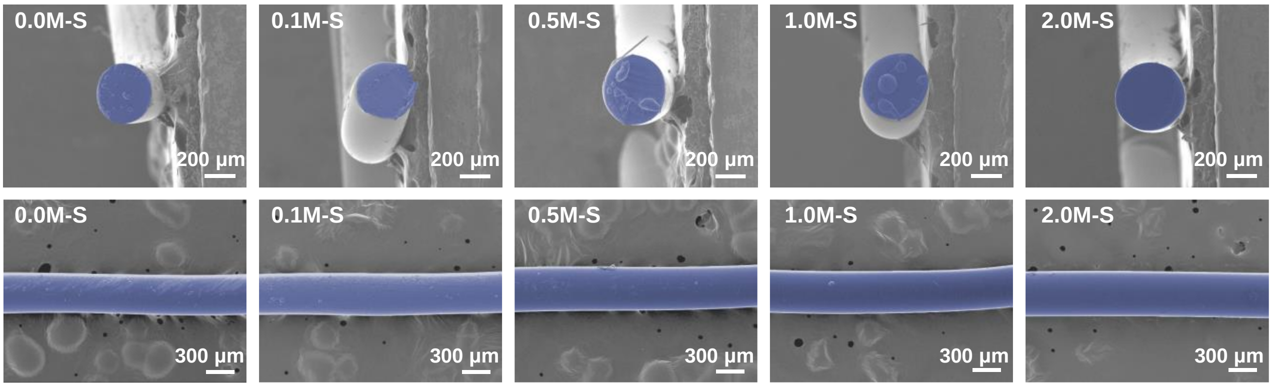

Supplementary Fig. 1 Scanning electron microscopy (SEM) images of silk fibroin-based ionic hydrogel fibers (SIH fibers).

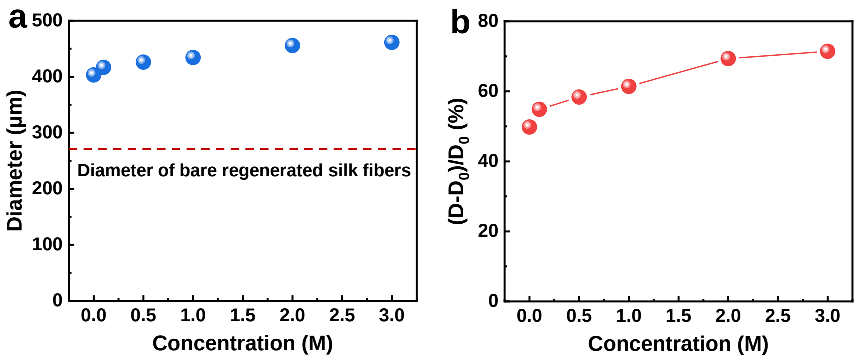

Supplementary Fig. 2 Diameters of SIH fibers. **a** Diameter of fibers in different groups. The red dashed line signifies the diameter of bare regenerated silk fibers (D_0=269 µm). **b** Diameter variations of the different fiber groups in comparison to bare regenerated silk fibers. D: diameter of SIH fibers.

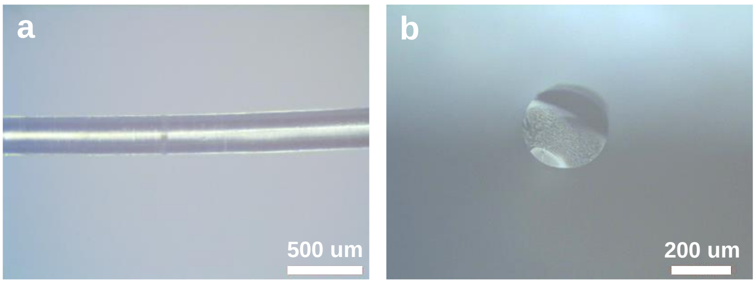

Supplementary Fig. 3 Morphology and dimensions of bare regenerated silk fibers. **a** Surface. **b** Cross-section.

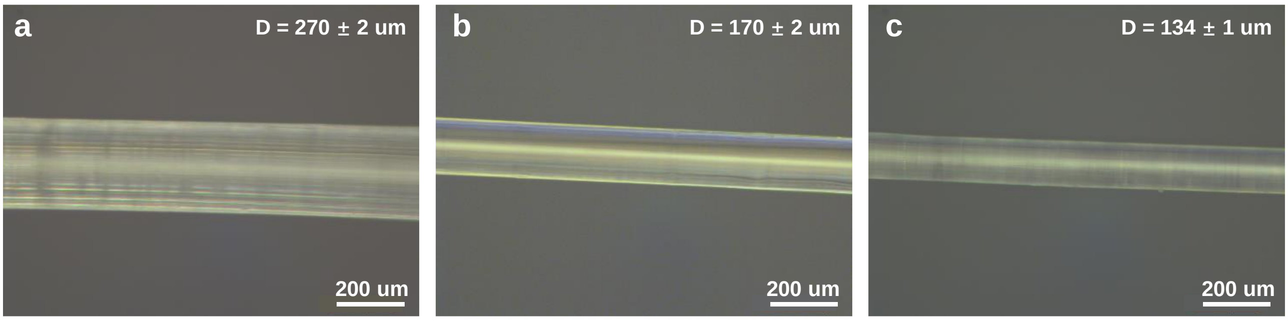

Supplementary Fig. 4 Optical images of SIFs prepared using spinnerets with different diameters and feeding rates. **a** 20 G, 240 μL·min^{-1}. **b** 22 G, 180 μL·min^{-1}. **c** 24 G, 140 μL·min^{-1}. D: Diameter of SIFs. The error bars show the standard deviation (*n*=5 for each group).

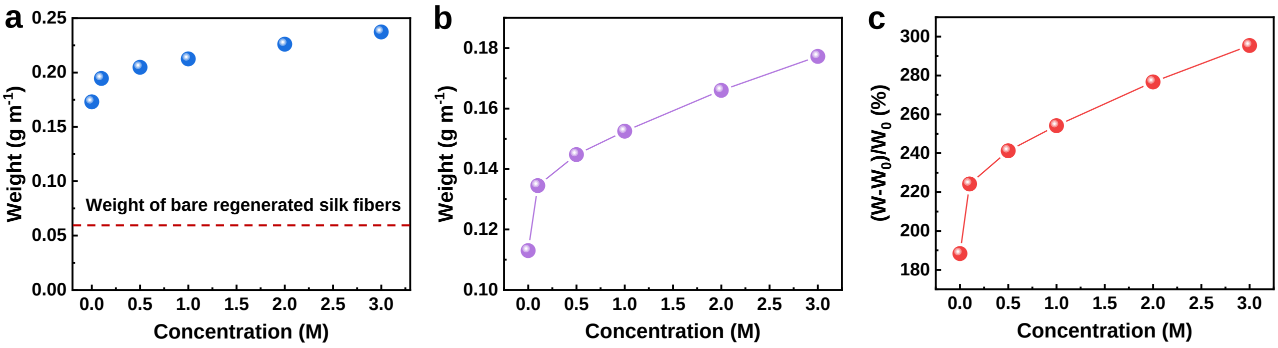

Supplementary Fig. 5 The weight change of silk fibers during the solvent process in ionic liquid tank. **a** Weight of fibers per unit length. The red dashed line represents the weight per unit length of bare regenerated silk (W_0=0.6 g·m^{-1}). **b** Solvent contents in unit-length fibers. **c** Weight variations of SIH fibers prepared with varied ionic liquid concentrations in comparison to bare regenerated silk. W: Weight of silk fibers.

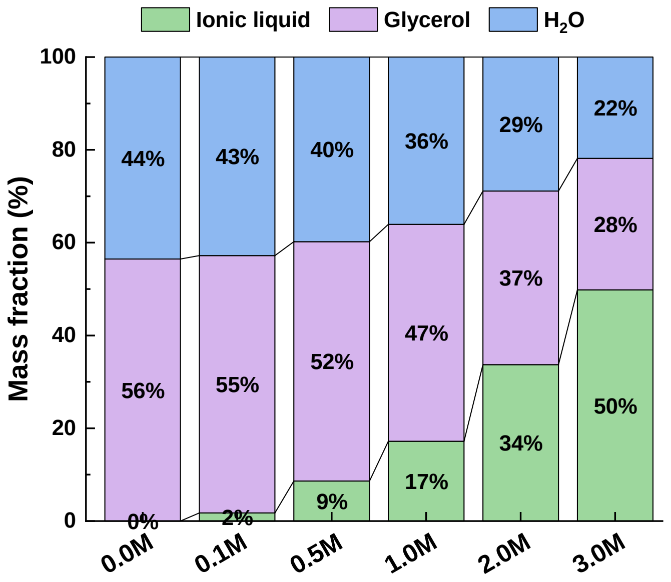

Supplementary Fig. 6 Component content in ionic liquid solutions with different concentrations

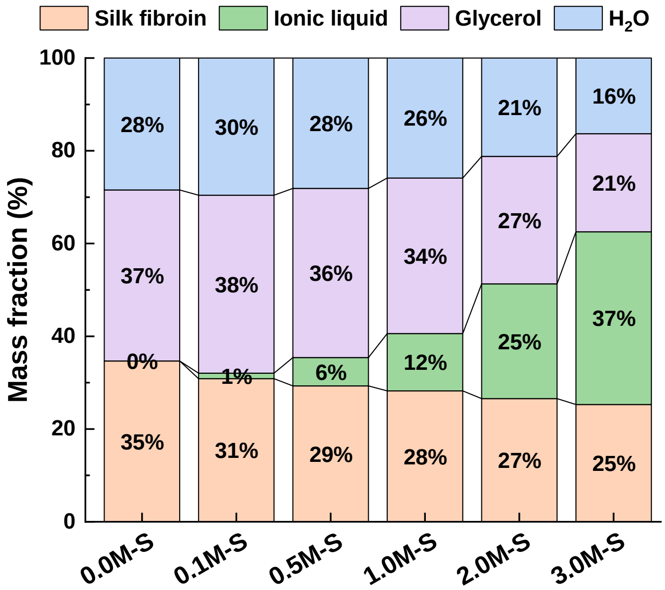

Supplementary Fig. 7 Contents of different components in various SIH fibers.

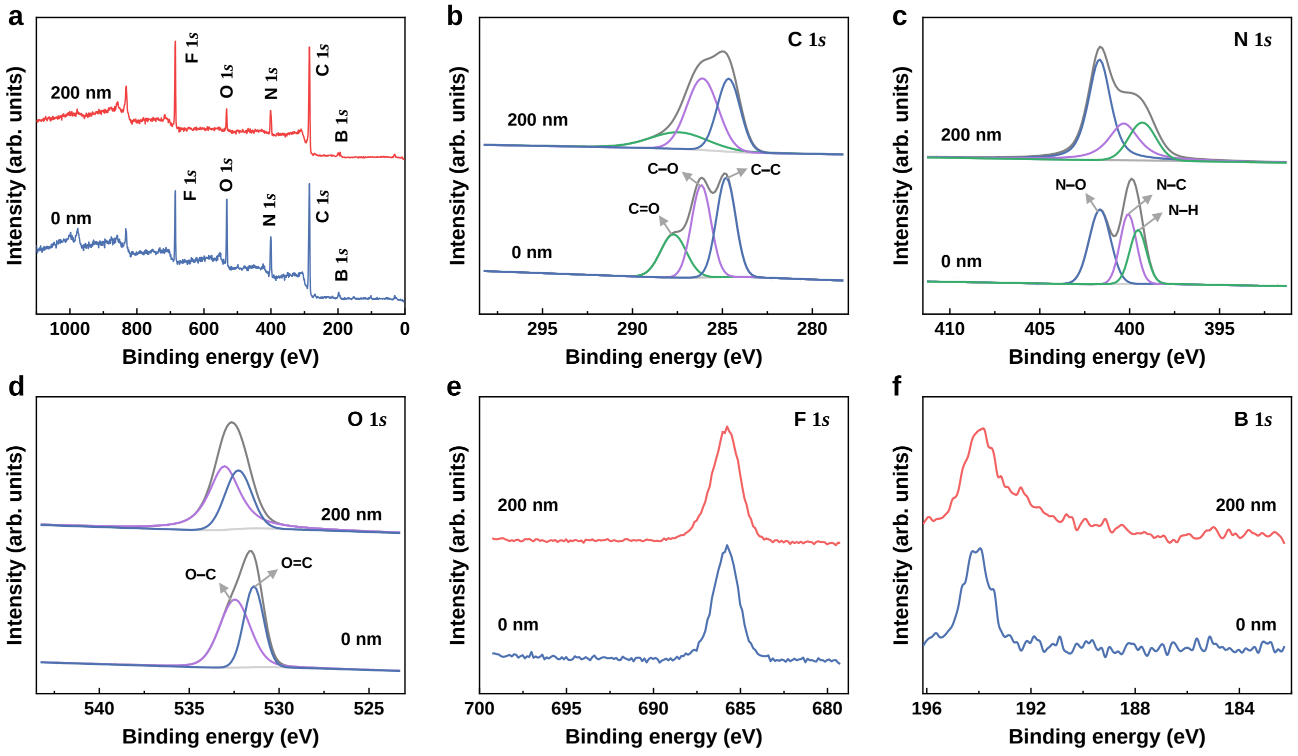

Supplementary Fig. 8 XPS analysis of the surface and inside (depth 200 nm) of 3.0 M-S. **a** XPS survey spectra of the fibers. **b-f** High resolution C 1*s* (**b**), N 1*s* (**c**), O 1*s* (**d**), F 1*s* (**e**), and B 1*s* (**f**) profiles of the fibers.

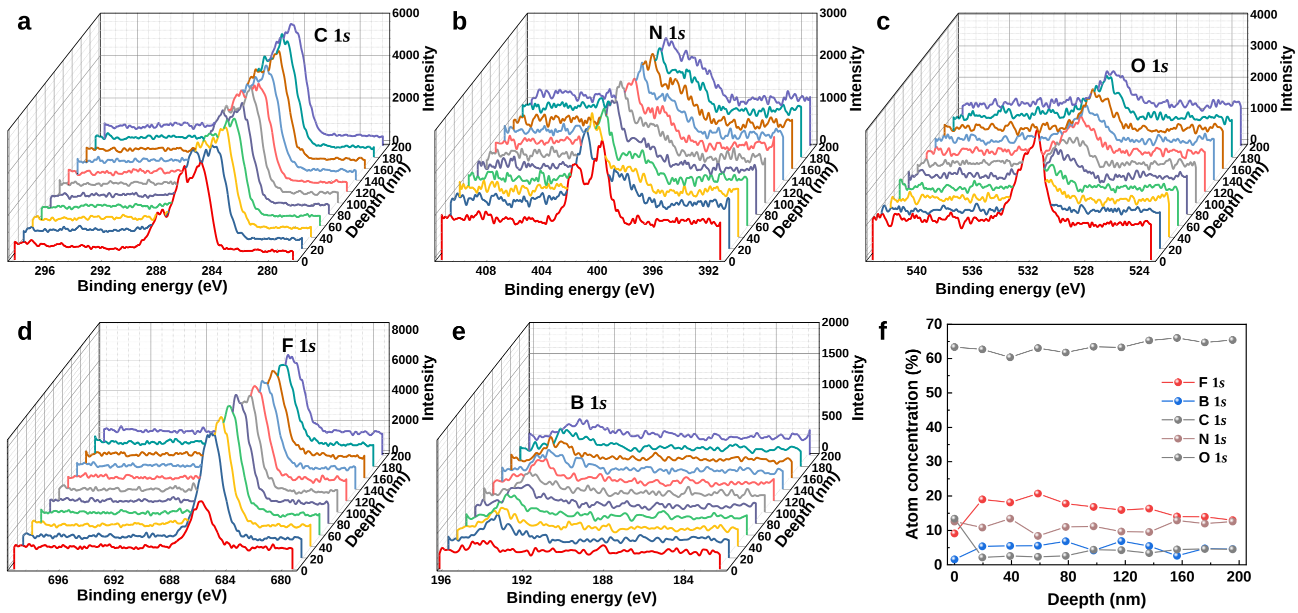

Supplementary Fig. 9 Depth profiling of XPS for 3.0 M-S. **a-e** XPS profile curves of C 1*s* (**a**), N 1*s* (**b**), O 1*s* (**c**), F 1*s* (**d**), and B 1*s* (**e**) for 3.0 M-S. **f** Atom concentration of C, N, O, F, and B in 3.0 M-S.

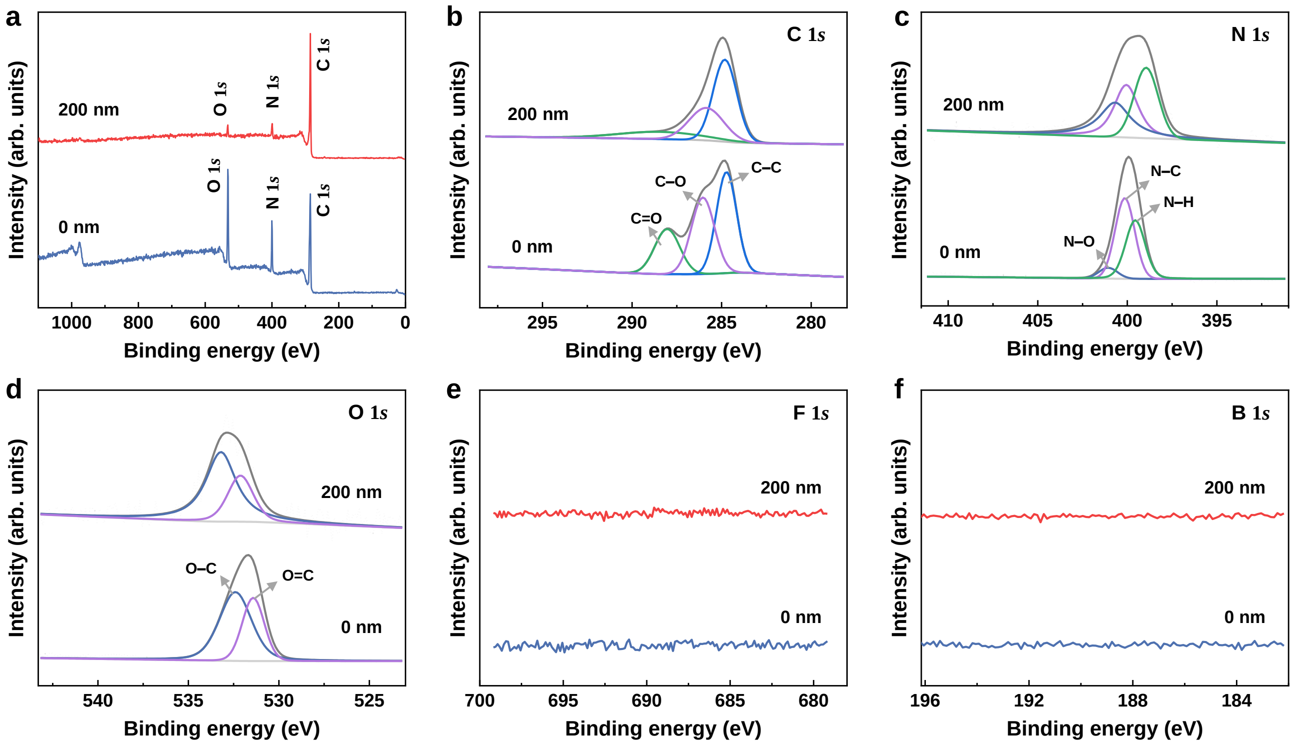

Supplementary Fig. 10 XPS analysis of the surface and inside (depth 200 nm) of 0.0 M-S. **a** XPS survey spectra of the fibers. **b-f** High resolution C 1*s* (**b**), N 1*s* (**c**), O 1*s* (**d**), F 1*s* (**e**), and B 1*s* (**f**) profiles of the fibers.

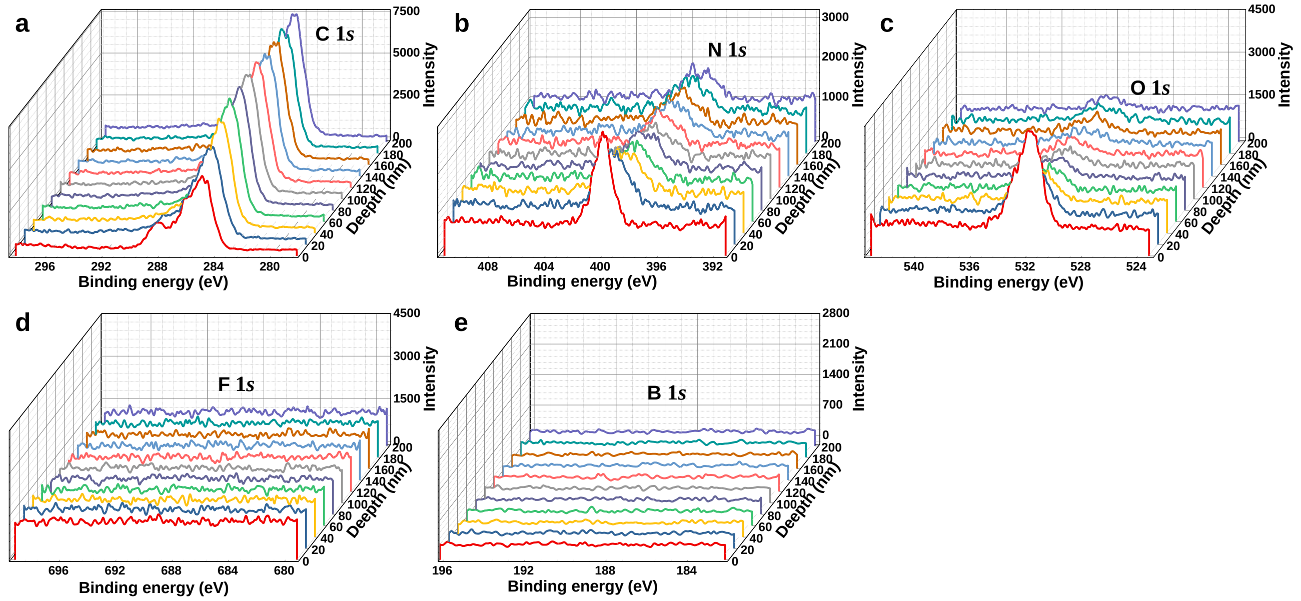

Supplementary Fig. 11 Depth profiling of XPS for 0.0 M-S. **a-e** XPS profile curves of C 1*s* (**a**), N 1*s* (**b**), O 1*s* (**c**), F 1*s* (**d**), and B 1*s* (**e**) for 0.0 M-S.

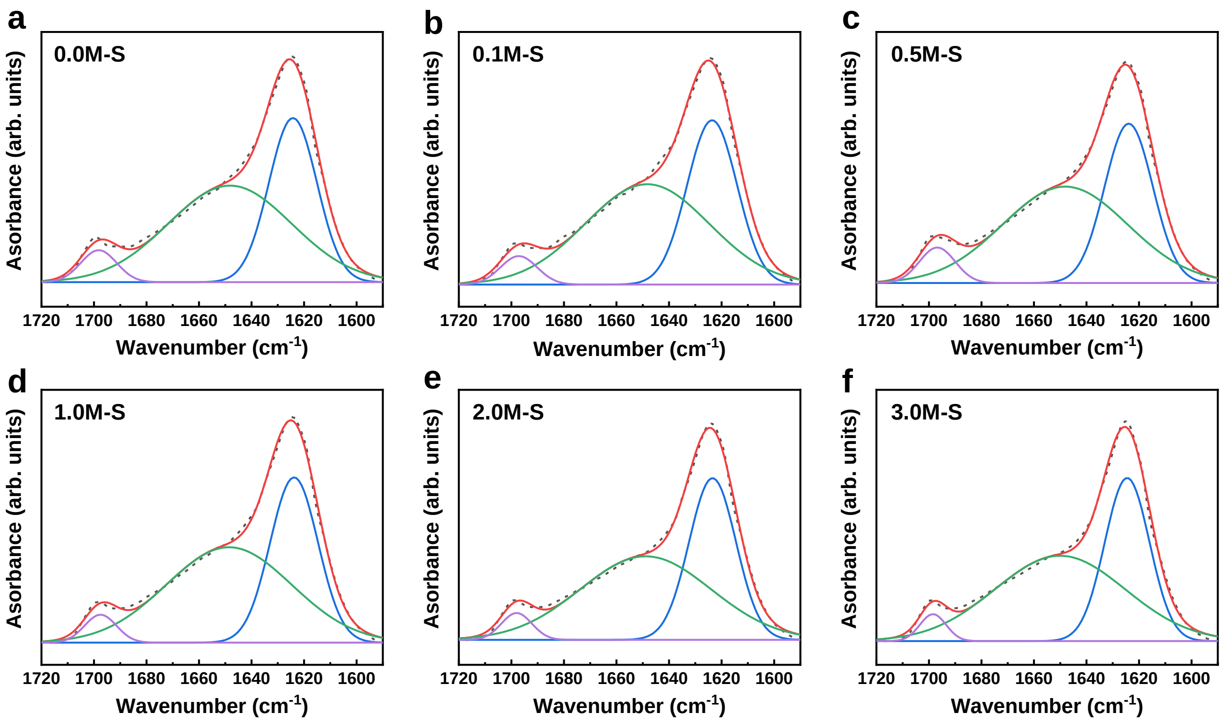

Supplementary Fig. 12 Examples of deconvolution of FTIR amide I band for different SIFs. The samples are 0.0M-S (**a**), 0.1M-S (**b**), 0.5M-S (**c**), 1.0M-S (**d**), 2.0M-S (**e**), and 3.0M-S (**f**), respectively.

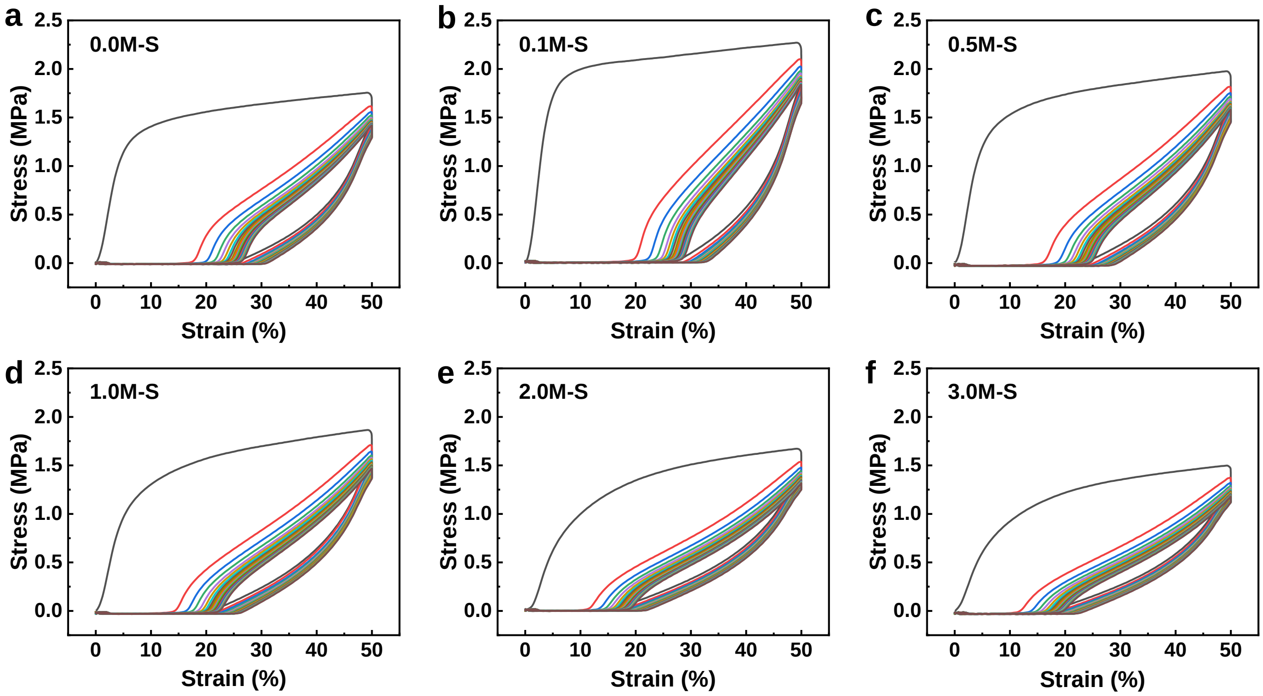

Supplementary Fig. 13 Cyclic tensile stress-strain curves with a tensile strain of 50% for twenty cycles of different SIH fiber samples. The samples are 0.0M-S (**a**), 0.1M-S (**b**), 0.5M-S (**c**), 1.0M-S (**d**), 2.0M-S (**e**), and 3.0M-S (**f**), respectively.

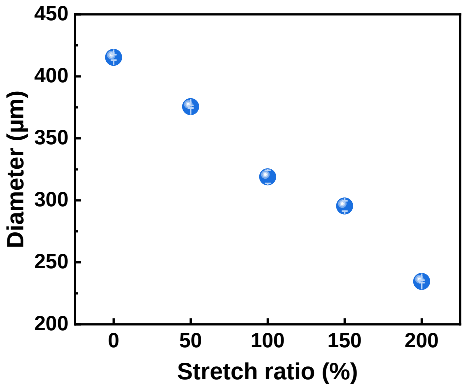

Supplementary Fig. 14 Diameter variations of SIH fibers with varied stretching ratios. The error bars show the standard deviation (*n*=3 for each group).

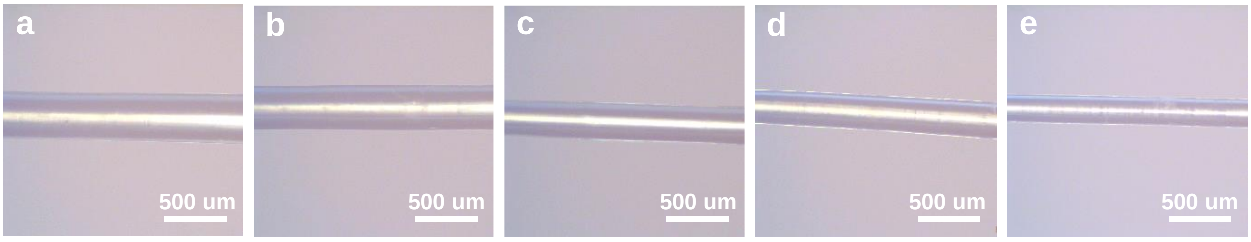

Supplementary Fig. 15 Optical images of SIH fibers prepared with varied stretching ratios. **a** 0%. **b** 50%. **c** 100%. **d** 150%. **e** 200%.

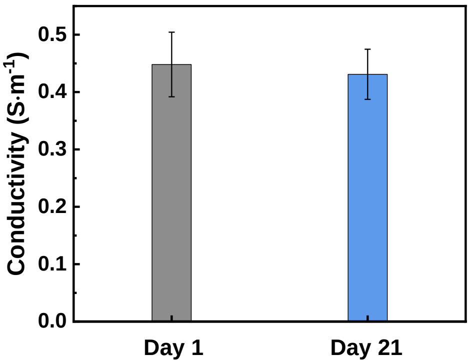

Supplementary Fig. 16 Conductivity comparison of SIFs (3.0M-S) before/after being stored at 20 °C and 43% humidity for 21 days. The error bars show the standard deviation (*n*=3 for each group).

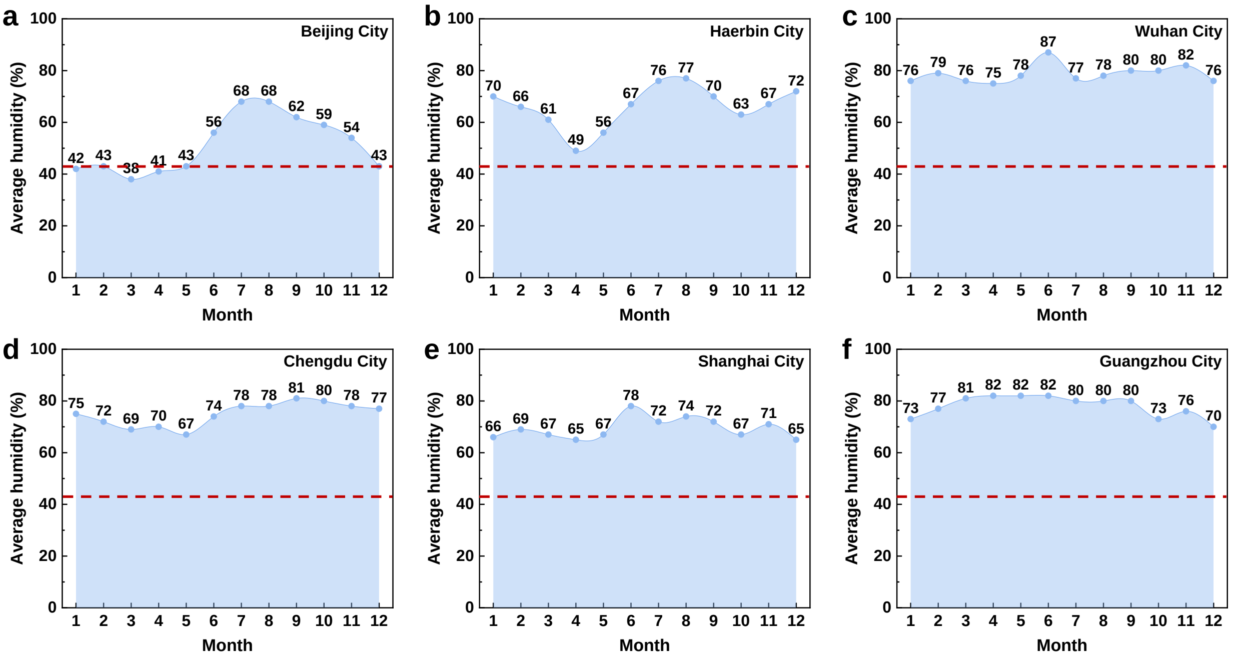

Supplementary Fig. 17 The monthly average humidity throughout the year of typical cities of China (2009-2018). **a** Beijing City. **b** Haerbin City. **c** Wuhan City. **d** Chengdu City. **e** Shanghai City. **f** Guangzhou City. The red dashed lines represent the humidity of 43%. (Data source: http://www.weather.com.cn)

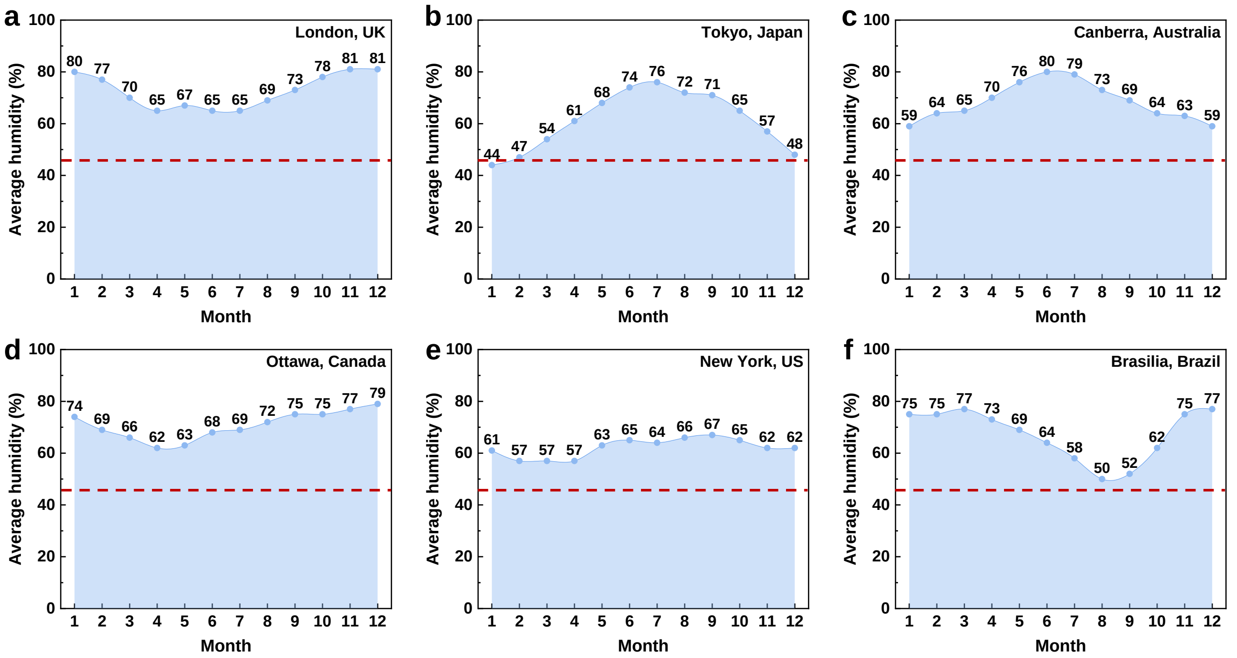

Supplementary Fig. 18. The monthly average humidity throughout the year of typical cities in the world (2005-2015 for London, 1985-2015 for other cities). **a** London, UK. **b** Tokyo, Japan. **c** Canberra, Australia. **d** Ottawa, Canada. **e** New York, US. **f** Brasilia, Brazil. The red dashed lines represent the humidity of 43%. (Data source: http://www.timeanddate.com/weather)

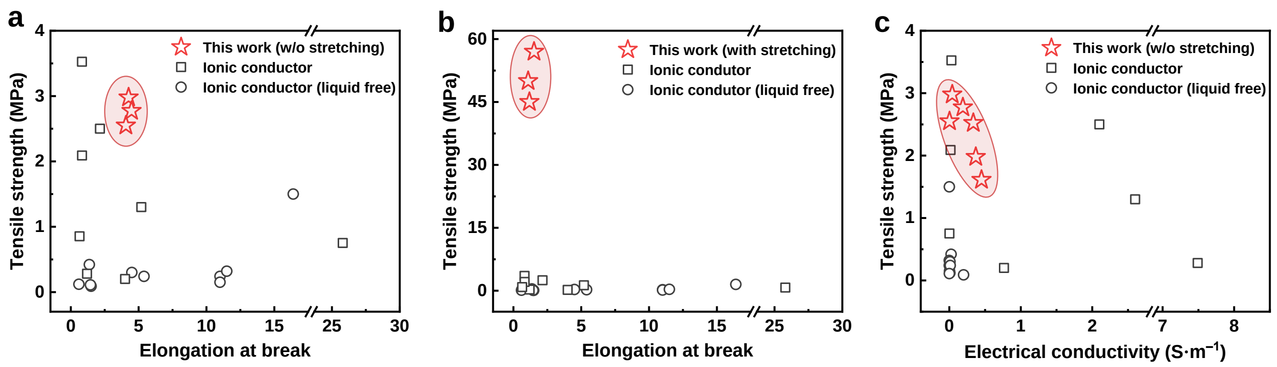

Supplementary Fig. 19 Performance comparison of SIH fibers with ionic conductors. **a** Mechanical comparison between SIH fibers and other ionic conductive materials. **b** Mechanical comparison between stretched SIH fibers and other ionic conductive materials. **c** Comparison of combined mechanical and electrical performance between SIH fibers and other ionic conductors.

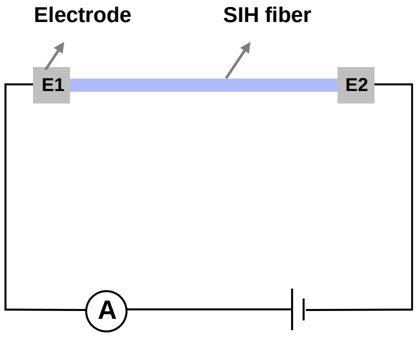

Supplementary Fig. 20 The circuit diagram of the perceptual textiles shown in Fig. 4a.

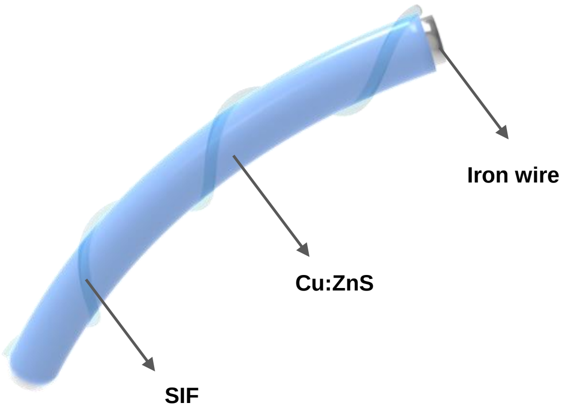

Supplementary Fig. 21 Illustration of the structure and composition of electroluminescent fibers in Fig. 1g.

Supplementary Tables 1 to 3

Supplementary Table 1 Composition of ionic liquid solutions with varied concentration

Solution	Weight fraction (%)		
	Ionic liquid	Glycerol	Water
0.0 M	0.0	56.5	43.5
0.1 M	1.7	55.5	42.8
0.5 M	8.6	51.6	39.8
1.0 M	17.2	46.8	36.1
2.0 M	33.7	37.4	28.9
3.0 M	49.8	28.3	21.8

Supplementary Table 2 Composition of SIH fibers fabricated with different concentrations of ionic liquid solutions.

Sample	Weight fraction (%)			
	Silk fibroin	Ionic liquid	Glycerol	Water
0.0 M-S	34.7	0.0	36.9	28.4
0.1 M-S	30.8	1.2	38.4	29.6
0.5 M-S	29.3	6.1	36.5	28.1
1.0 M-S	28.2	12.3	33.6	25.9
2.0 M-S	26.5	24.8	27.5	21.2
3.0 M-S	25.3	37.2	21.2	16.3

Supplementary Table 3 The mechanical and electrical properties of ionic conductors in Supplementary Fig. 13

Name	Tensile strength (MPa)	Elongation at break	Conductivity ($S \cdot m^{-1}$)	References
Ionic conductor (Liquid free)	0.09	1.5	0.2	[1]
	0.42	1.37	0.025	[2]
	0.24	11	1E-07	[3]
	0.15	11	0.0024	[4]
	0.12	0.6	0.00801	[5]
	0.32	11.5	0.000001	[6]
	0.3	4.5	0.01	[7]
	0.24	5.4	0.0131	[8]
	0.11	1.45	0.001	[9]
	1.5	16.4	0.001	[10]
Ionic conductor	1.3	5.2	2.6	[11]
	0.752	25.8	0.00169	[12]
	0.28	1.2	7.49	[13]
	0.2	4	0.765	[14]
	2.5	2.15	2.1	[15]
	3.525	0.8233	0.03	[16]
	2.09	0.8308	0.017	
	0.854	0.6466	-	

Supplementary references

1 Li, R. a., Chen, G., He, M., Tian, J. & Su, B. Patternable transparent and conductive elastomers towards flexible tactile/strain sensors. *J. Mater. Chem. C* **5**, 8475-8481 (2017).

2 Lopez, J. *et al.* A dual-crosslinking design for resilient lithium-ion conductors. *Adv. Mater.* **30**, 1804142 (2018).

3 Shi, L. *et al.* Highly stretchable and transparent ionic conducting elastomers. *Nat. Commun.* **9**, 2630 (2018).

4 Dang, C. *et al.* Transparent, highly stretchable, rehealable, sensing, and fully recyclable ionic conductors fabricated by one-step polymerization based on a small biological molecule. *Adv. Funct. Mater.* **29**, 1902467 (2019).

5 Zhou, B. *et al.* Self-healing composite polymer electrolyte formed via supramolecular networks for high-performance lithium-ion batteries. *J. Mater. Chem. A* **7**, 10354-10362 (2019).

6 Zhang, P. *et al.* Stretchable, transparent, and thermally stable triboelectric nanogenerators based on solvent-free ion-conducting elastomer electrodes. *Adv. Funct. Mater.* **30**, 1909252 (2020).

7 Li, R. a. *et al.* Autonomous self-healing, antifreezing, and transparent conductive elastomers. *Chem. Mater.* **32**, 874-881 (2020).

8 Qu, X. *et al.* Solid-state and liquid-free elastomeric ionic conductors with autonomous self-healing ability. *Mater. Horiz.* **7**, 2994-3004 (2020).

9 Kim, H. J., Chen, B., Suo, Z. & Hayward, R. C. Ionoelastomer junctions between polymer networks of fixed anions and cations. *Science* **367**, 773-776 (2020).

10 Yiming, B. *et al.* A mechanically robust and versatile liquid-free ionic conductive elastomer. *Adv. Mater.* **33**, 2006111 (2021).

11 Zhou, Y. *et al.* Highly stretchable, elastic, and ionic conductive hydrogel for artificial soft electronics. *Adv. Funct. Mater.* **29**, 1806220 (2019).

12 Yiming, B. *et al.* Ambiently and mechanically stable ionogels for soft ionotronics. *Adv. Funct. Mater.* **31**, 2102773 (2021).

13 Zhang, X.-F. *et al.* Inorganic salts induce thermally reversible and anti-freezing cellulose hydrogels. *Angew. Chem. Int. Edit.* **58**, 7366-7370 (2019).

14 Song, J. *et al.* Mechanically and electronically robust transparent organohydrogel fibers. *Adv.*

Mater. **32**, 1906994 (2020).

15 Li, M. *et al.* Wearable and robust polyimide hydrogel fiber textiles for strain sensors. *ACS Appl. Mater. Inter.* **13**, 43323-43332 (2021).

16 Zhang, M. *et al.* Spinning continuous high-strength bacterial cellulose hydrogel fibers for multifunctional bioelectronic interfaces. *J. Mater. Chem. A* **9**, 12574-12583 (2021).
