# Supplementary material for: Intelligent perceptual textiles based on ionic-conductive and strong silk fibers
Source: Nat Commun. 2024 Apr 17;15:3289. doi: 10.1038/s41467-024-47665-y (PMC11024123; doi:10.1038/s41467-024-47665-y)
Supplement: Supplementary file 3 — Description of Additional Supplementary Files [file 41467_2024_47665_MOESM3_ESM.pdf]

Description of Additional Supplementary Files

Supplementary Movie Legends:

Supplementary Movie 1: A display device with SIH fibers as flexible and transparent electrodes

Supplementary Movie 2: Controlling of a robot hand by touching a SIH fiber-based textile.
